# The liver–gut–brain axis in metabolic dysfunction-associated steatotic liver disease: bidirectional communication, clinical implications, and future perspectives

**DOI:** 10.3389/fendo.2026.1911290

**Published:** 2026-08-13

**Authors:** Yulian Ren, Dongqing Hu, Lu Li, Lei Wang, Xianqing Meng

**Affiliations:** 1 Department of Hepatobiliary Medicine, Affiliated Hospital of Shandong University of Traditional Chinese Medicine, Jinan, China; 2 Infection Control Office, Affiliated Hospital of Shandong University of Traditional Chinese Medicine, Jinan, China

**Keywords:** metabolic dysfunction-associated steatotic liver disease, MASLD, liver–gut–brain axis, gut microbiota, intestinal barrier, neuroinflammation, biomarkers

## Abstract

**Background:**

Metabolic dysfunction-associated steatotic liver disease (MASLD) affects approximately one-third of adults worldwide and is increasingly recognized as a multisystem disorder. In addition to hepatic and cardiometabolic complications, many patients report fatigue, depression, anxiety, sleep disturbance, and subjective cognitive complaints. These manifestations are clinically important but correlate inconsistently with liver histology and may arise from several interacting mechanisms.

**Aim:**

To critically synthesize evidence on the liver–gut–brain axis in MASLD, distinguish established pathways from indirect or preclinical hypotheses, and evaluate implications for biomarkers, patient assessment, and treatment.

**Methods:**

PubMed was searched through July 15, 2026 using combinations of terms related to MASLD/MASH, intestinal barrier function, gut microbiota, autonomic and stress signaling, hepatic inflammation and fibrosis, neuroinflammation, cognition, biomarkers, and treatment. Guidelines, randomized trials, prospective human studies, systematic reviews, and mechanistically informative experimental studies were prioritized. Evidence derived from cirrhosis, hepatic encephalopathy, other diseases, or animal models is explicitly identified.

**Results:**

The axis comprises neural, endocrine, immune, and metabolic communication. Stress-related hypothalamic–pituitary–adrenal and sympathetic activation may influence behavior, adipose tissue, intestinal motility, mucosal immunity, and epithelial integrity. Barrier dysfunction can increase portal exposure to microbial products that activate hepatic inflammatory pathways and aggravate insulin resistance. Conversely, hepatic and intestinal cytokines, bile acids, hepatokines, and microbial metabolites may signal at vascular and neural interfaces, although direct causal evidence for brain effects in noncirrhotic MASLD remains limited. Lifestyle and cardiometabolic risk management remain foundational. Resmetirom and semaglutide have regulatory indications for selected adults with noncirrhotic MASH and moderate-to-advanced fibrosis, whereas microbiota-directed therapies and neuromodulation remain investigational.

**Conclusion:**

The liver–gut–brain axis is a useful integrative framework, but its component pathways have unequal evidentiary support. Longitudinal human studies using standardized hepatic, microbial, inflammatory, autonomic, and neurobehavioral outcomes are required before axis-based biomarkers or endotypes can guide routine care.

## 1 Introduction: MASLD as a multisystem disorder

Metabolic dysfunction-associated steatotic liver disease (MASLD) is the consensus term for steatotic liver disease occurring in the presence of at least one cardiometabolic risk factor and without another dominant etiology. The inflammatory phenotype is termed metabolic dysfunction-associated steatohepatitis (MASH). These terms replace the historical labels non-alcoholic fatty liver disease (NAFLD) and non-alcoholic steatohepatitis (NASH), respectively (1, 2). Throughout this review, MASLD and MASH are preferred. NAFLD and NASH are retained only for historical study populations, trial names, endpoints, or diagnostic criteria. Metabolic dysfunction-associated fatty liver disease (MAFLD) is a distinct diagnostic framework and is identified explicitly when an original study used that definition.

MASLD affects approximately one-third of the global adult population, although prevalence varies by region, sampling method, and diagnostic modality (3). Its established pathophysiology involves adipose tissue dysfunction, systemic and hepatic insulin resistance, increased fatty-acid delivery and *de novo* lipogenesis, lipotoxicity, mitochondrial and endoplasmic reticulum stress, hepatocyte injury, immune activation, and progressive fibrosis (4, 5). Current guidelines consequently emphasize cardiometabolic risk reduction and staged assessment for advanced fibrosis (6, 7).

This hepatic framework does not fully account for the heterogeneity of patient experience. Fatigue, depression, anxiety, sleep disturbance, and cognitive complaints are common and can substantially impair quality of life (8). Their relationship to steatosis, inflammatory activity, or fibrosis is inconsistent. These symptoms may reflect obesity, diabetes, sleep apnea, vascular disease, medication exposure, socioeconomic adversity, or primary psychiatric and neurological disorders in addition to possible inter-organ signaling (9). Accordingly, symptom burden and fibrosis risk should be assessed as related but non-interchangeable dimensions of MASLD.

The liver–gut–brain axis provides an integrative framework for examining these relationships (10–12). It encompasses neural communication through vagal and autonomic pathways; endocrine signaling through stress hormones, gut peptides, bile acids, adipokines, and hepatokines; immune signaling through cytokines and microbial-associated molecular patterns; and metabolic signaling through microbial and host-derived metabolites (13, 14). The framework is biologically plausible, but evidence ranges from direct human observations to extrapolation from cirrhosis, neurological disease, and experimental models. Anatomical connectivity or cross-sectional association alone does not establish a causal organ-to-organ pathway.

Within the proposed top-down pathway, chronic stress and adverse health behaviors may alter autonomic output, intestinal motility, mucosal immunity, epithelial integrity, and systemic metabolism. Increased permeability may enhance portal exposure to microbial products and amplify hepatic inflammation. In the bottom-up direction, a steatotic and inflamed liver can modify the circulating cytokine, bile acid, lipid, and hepatokine milieu. These signals may influence cerebrovascular endothelium, peripheral nerves, and neuroimmune pathways, but several liver-to-brain mechanisms remain unproven in noncirrhotic MASLD. The gut microbiota may amplify both directions because it responds to diet, medications, stress physiology, bile acids, intestinal transit, and inflammation while modifying metabolite production.

This narrative review first defines the structural and functional basis of the axis, with particular emphasis on intestinal barrier biology and the integration of hepatic stellate cells (HSCs) into broader hepatic pathophysiology. It then examines the proposed top-down and bottom-up pathways and the microbiota as a context-dependent amplifier. Finally, it evaluates methodological advances, candidate biomarkers, and therapeutic strategies, explicitly separating established MASLD management from investigational axis-targeted approaches.

What this review adds. Unlike reviews focused primarily on the gut–liver axis or the microbiota–brain axis, this review adds three elements: it integrates top-down neuroendocrine and behavioral influences with bottom-up hepatic, immune, and metabolic signaling in a single bidirectional MASLD model; it places intestinal barrier dysfunction and hepatic stellate cell activation within the established multicellular pathways of MASH and fibrosis; and it translates this framework into evidence-graded biomarker and therapeutic priorities, distinguishing clinically actionable care from investigational axis-targeted strategies.

## 2 Structural, functional, and cellular foundations of the liver–gut–brain axis

**Figure 1:**
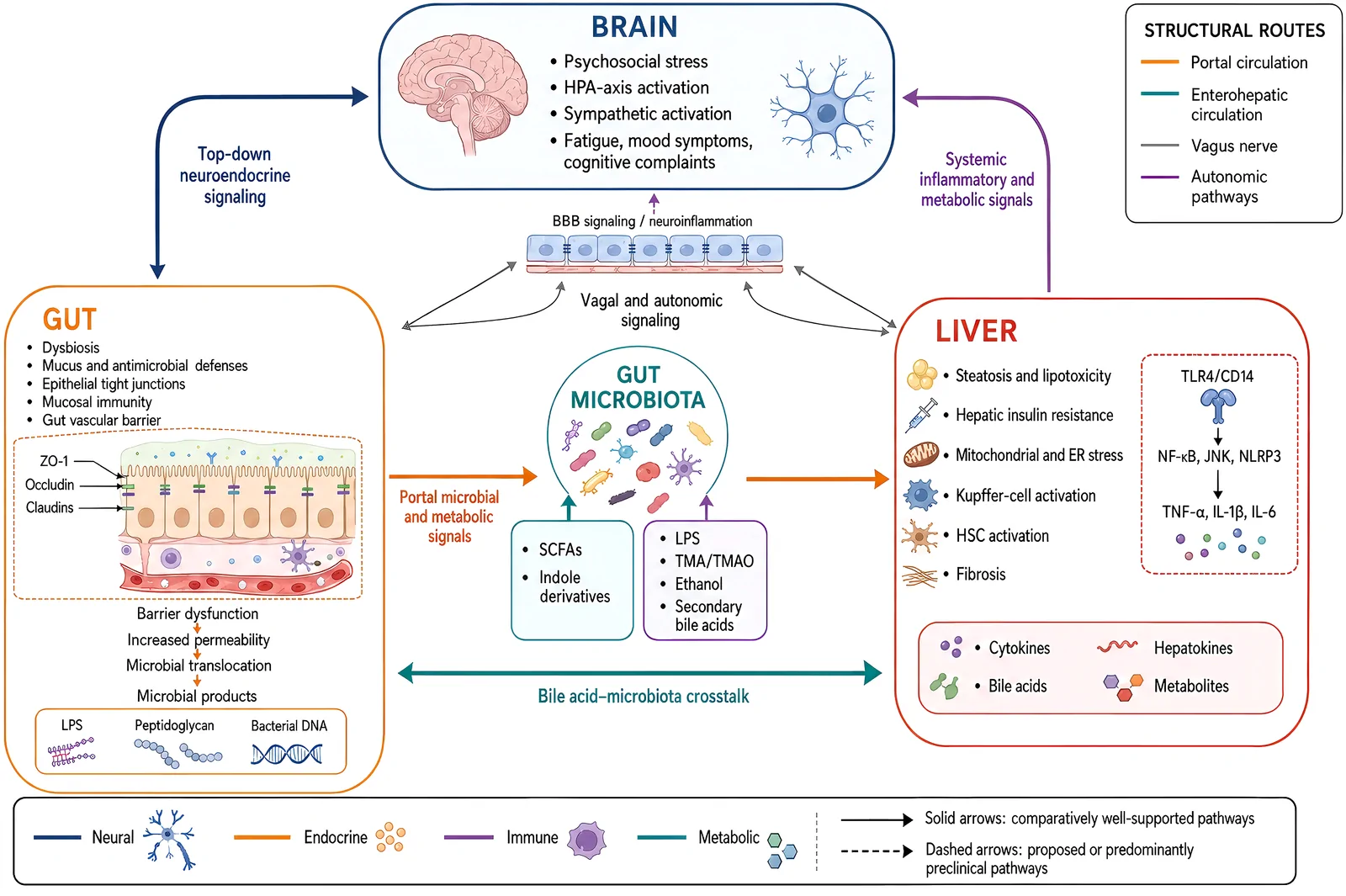
Structural and functional organization of the liver–gut–brain axis in metabolic dysfunction-associated steatotic liver disease. The brain, intestine, and liver communicate bidirectionally through neural, endocrine, immune, and metabolic pathways. Stress-related HPA-axis and sympathetic activation may alter intestinal motility, mucosal immunity, microbial composition, and epithelial barrier integrity. Disruption of mucus and antimicrobial defenses, epithelial tight junctions, mucosal immunity, and the gut vascular barrier increases permeability and portal exposure to LPS, peptidoglycan, and bacterial DNA. Microbial dysbiosis modifies SCFAs, indole derivatives, TMA/TMAO, ethanol, and secondary bile acids. In the liver, these signals can activate TLR4/CD14, NF-κB/JNK, and NLRP3-associated pathways, contributing to cytokine production, insulin resistance, steatosis, lipotoxicity, Kupffer-cell activation, HSC activation, and fibrosis. Liver- and gut-derived cytokines, bile acids, hepatokines, and metabolites may influence BBB signaling and neuroinflammation. Portal and enterohepatic circulation provide major gut–liver routes, while vagal and autonomic pathways contribute to neural communication. Solid arrows indicate comparatively well-supported pathways; dashed arrows indicate proposed, indirect, or predominantly preclinical mechanisms. BBB, blood–brain barrier; CD14, cluster of differentiation 14; DNA, deoxyribonucleic acid; ER, endoplasmic reticulum; HPA, hypothalamic–pituitary–adrenal; HSC, hepatic stellate cell; IL-1β, interleukin-1 beta; IL-6, interleukin-6; JNK, c-Jun N-terminal kinase; LPS, lipopolysaccharide; MASLD, metabolic dysfunction-associated steatotic liver disease; NF-κB, nuclear factor kappa B; NLRP3, NOD-, LRR- and pyrin domain-containing protein 3; SCFAs, short-chain fatty acids; TLR4, Toll-like receptor 4; TMA, trimethylamine; TMAO, trimethylamine N-oxide; TNF-α, tumor necrosis factor alpha; ZO-1, zonula occludens-1.

The liver–gut–brain axis comprises overlapping anatomical routes and signaling systems rather than a single communication channel. Portal and systemic circulation, enterohepatic bile acid cycling, autonomic nerves, vascular interfaces, immune cells, and microbial metabolism operate on different time scales and with different degrees of specificity. This architecture is summarized in **Figure 1** after the principal signaling modalities are described.

### 2.1 Anatomical communication routes

#### 2.1.1 Portal and systemic circulation

The portal venous system is the most direct anatomical connection between the intestine and liver. It delivers nutrients together with microbial products and metabolites, while the liver provides first-pass metabolic and immunological surveillance (15, 16). Persistent exposure to lipopolysaccharide (LPS), peptidoglycan, bacterial DNA, or other microbial-associated molecular patterns can activate hepatic innate immune pathways when epithelial, vascular, and hepatic containment mechanisms are insufficient (17, 18). Portal transport is not uniformly injurious: enterically derived high-density lipoprotein can bind LPS and limit liver injury, illustrating endogenous protection within this route (15). Liver-derived proteins, cytokines, extracellular-matrix fragments, lipoproteins, and endocrine factors subsequently enter systemic blood and can signal to distant organs.

#### 2.1.2 Enterohepatic bile acid circulation

Bile acids are synthesized in the liver, secreted into the intestine, and largely reabsorbed in the terminal ileum. Microbial deconjugation and transformation modify the pool before it returns through the portal circulation (19, 20). Signaling through farnesoid X receptor (FXR), fibroblast growth factor 19, and Takeda G protein-coupled receptor 5 influences bile acid synthesis, lipid and glucose metabolism, inflammation, intestinal barrier function, and microbial ecology (21, 22). The consequences are context dependent: primary and secondary bile acids differ in hydrophobicity, receptor activity, concentration, and tissue distribution. They should not be described as uniformly protective or uniformly neurotoxic (23).

#### 2.1.3 Vagal and sympathetic pathways

Vagal afferents convey visceral, nutrient, and inflammatory information to brainstem nuclei, whereas efferent autonomic output influences gastrointestinal motility, secretion, immune tone, pancreatic function, and hepatic metabolism (24, 25). Hepatic neural regulation frequently involves indirect circuits through the portal region, gut, pancreas, brainstem, and sympathetic ganglia rather than a single dedicated liver-to-brain nerve. Vagal signaling demonstrated in other inflammatory or metabolic settings therefore cannot be assumed to have the same magnitude or therapeutic relevance in MASLD (14).

### 2.2 Functional signaling modalities

Four interacting modalities organize communication. Neural signaling operates through autonomic afferent and efferent pathways. Endocrine signaling includes glucocorticoids, catecholamines, glucagon-like peptide 1, glucose-dependent insulinotropic polypeptide, peptide YY, bile acids, adipokines, and hepatokines. Immune signaling involves cytokines, chemokines, complement, and microbial-associated molecular patterns. Metabolic signaling includes short-chain fatty acids (SCFAs), indole derivatives, kynurenines, trimethylamine-related metabolites, endogenous ethanol, phenylacetylglutamine, and host lipid species. These categories overlap; for example, bile acids and SCFAs have metabolic, endocrine, immune, and neural effects.

SCFAs, particularly butyrate, support colonocyte energy metabolism, epithelial integrity, and context-dependent immune regulation (26, 27). Tryptophan can enter microbial indole, host serotonin, or kynurenine pathways, each with different effects on barrier function, immunity, vascular biology, and neural activity (28). Trimethylamine N-oxide and phenylacetylglutamine have been associated with cardiometabolic and neurovascular phenotypes (29), whereas other microbial metabolites have been linked to cognitive aging (30). Associations depend on diet, renal clearance, medication, receptor expression, and analytical platform and should not be interpreted automatically as causal.

The cholinergic anti-inflammatory pathway offers an experimental mechanism by which vagal activity can restrain macrophage cytokine production. Acetylcholine-sensitive pathways can modify macrophage polarization and migration (31, 32). However, the anatomical implementation and effect size vary among organs, and evidence for a clinically relevant hepatic vagal anti-inflammatory response in MASLD remains incomplete. Likewise, circulating cytokines need not cross an intact blood–brain barrier (BBB) in large quantities; they may signal through endothelial cells, circumventricular organs, meningeal or perivascular immune cells, and peripheral sensory nerves (25).

Endocrine communication extends beyond stress hormones. Intestinal glucagon-like peptide 1, glucose-dependent insulinotropic polypeptide, peptide YY, and cholecystokinin influence appetite, gastric emptying, insulin secretion, and vagal afferent activity. The liver contributes fibroblast growth factor 21, fetuin-A, insulin-like growth factor 1, and other hepatokines, while adipose tissue supplies leptin, adiponectin, free fatty acids, and inflammatory mediators. These factors act on several tissues and are strongly modified by obesity and diabetes. Consequently, a circulating concentration cannot be assigned to a unidirectional liver-to-brain or gut-to-liver pathway without source tracing, tissue-specific perturbation, or rigorous mediation analysis. Endocrine and metabolic effects should also be distinguished from pharmacological receptor activation, which may produce concentrations and tissue distributions not encountered physiologically.

The structural routes and signaling modalities are integrated in **Figure 1**.

### 2.3 Intestinal barrier dysfunction as a central interface

The intestinal barrier comprises several interacting layers: epithelial cells connected by claudins, occludin, and zonula occludens proteins; mucus and antimicrobial peptides; mucosal immune structures; commensal microbial communities; and the gut vascular barrier (33, 34). Together, these layers permit nutrient exchange while restricting viable organisms and microbial products. Barrier function is therefore not equivalent to a single tight-junction protein, a stool taxonomic profile, or the nonspecific concept of a uniformly ‘leaky gut.’

In MASLD, saturated-fat-rich diets, obesity, altered bile acid signaling, dysbiosis, low-grade mucosal inflammation, medications, and neuroendocrine stress may impair mucus production, epithelial repair, tight-junction organization, and vascular containment (35, 36). Increased permeability enhances portal exposure to LPS, peptidoglycan, and bacterial DNA. These ligands engage CD14/Toll-like receptor 4 and other pattern-recognition receptors on Kupffer cells, hepatocytes, endothelial cells, and HSCs. Downstream NF-κB, c-Jun N-terminal kinase, and inflammasome signaling promotes cytokine release, insulin resistance, and fibrogenesis (37–39). Experimental intestinal transmembrane 6 superfamily member 2 (TM6SF2) manipulation provides direct mechanistic evidence that intestinal lipid handling can influence steatohepatitis, although its effect size in heterogeneous human MASLD remains uncertain (40).

Human assessment requires complementary methods. Sugar permeability tests estimate regional paracellular transport; intestinal fatty acid-binding protein reflects enterocyte injury; LPS-binding protein and soluble CD14 reflect host responses to microbial exposure; and bacterial DNA assays require rigorous contamination control. Peripheral concentrations may differ from portal exposure because of hepatic clearance. Recent MASLD cohorts support associations among permeability, microbial translocation, steatosis, or fibrosis (41, 42), but antibiotics, proton-pump inhibitors, metformin, diabetes, alcohol, diet, geography, stool consistency, and sample handling can confound results (43). Barrier dysfunction is therefore an important modifier in a subset of patients, not a proven universal initiating event.

The gut vascular barrier adds a further checkpoint between the lamina propria and portal blood. Endothelial junctions, pericytes, immune cells, and extracellular matrix restrict dissemination after material crosses the epithelium. Mucosal immunoglobulin A, antimicrobial peptides, goblet-cell mucus, epithelial oxygen consumption, and colonization resistance also shape microbial proximity to the mucosa. Bile acids and SCFAs can support or disrupt these layers depending on species, concentration, receptor, and disease context. Barrier failure is therefore better conceptualized as a graded, multi-compartment process than as an all-or-none epithelial defect. This distinction explains why stool composition, serum microbial products, enterocyte-injury markers, and permeability tests may disagree within the same patient and why improvement in one readout does not necessarily demonstrate restoration of the complete barrier.

### 2.4 Integrated hepatic pathophysiology and fibrogenic responses

The hepatic component of the axis must remain anchored in established MASLD biology. Adipose tissue insulin resistance increases lipolysis and fatty-acid delivery, while hyperinsulinemia and nutrient excess stimulate hepatic *de novo* lipogenesis. Lipid species that exceed storage and export capacity induce lipotoxicity, mitochondrial dysfunction, endoplasmic reticulum stress, oxidative injury, and hepatocyte ballooning. Injured hepatocytes release danger signals that activate Kupffer cells and recruit monocyte-derived macrophages. Endothelial dysfunction, ductular reactions, adaptive immune responses, and extracellular-matrix remodeling then influence progression from steatosis to MASH and fibrosis (4, 5, 44, 45).

Macrophage states are dynamic rather than a simple M1/M2 dichotomy. Resident and recruited macrophages can produce TNF-α, IL-1β, IL-6, chemokines, reactive oxygen species, and profibrogenic mediators, but they also participate in efferocytosis, matrix degradation, and resolution (46, 47). HSCs are central effectors of fibrosis within this multicellular network. Signals from injured hepatocytes, macrophages, endothelial cells, platelets, adipokines, bile acids, and transforming growth factor beta (TGF-β) induce their transition from quiescent vitamin A-storing cells to proliferative, contractile, extracellular-matrix-producing myofibroblasts (48–50).

Hepatocyte adaptation and failure occur along a continuum. Triglyceride storage can initially buffer potentially toxic fatty acids, whereas diacylglycerols, ceramides, free cholesterol, and oxidized phospholipids can interfere with insulin signaling and organelle function. Persistent substrate excess alters mitochondrial beta-oxidation, respiratory-chain activity, redox balance, and mitochondrial quality control. The unfolded-protein response may initially restore endoplasmic reticulum homeostasis but can become inflammatory or proapoptotic when stress persists. Ballooned hepatocytes, apoptotic bodies, and damage-associated molecular patterns then recruit and reprogram immune cells. Liver sinusoidal endothelial capillarization and reduced nitric oxide further disturb perfusion and paracrine signaling. These processes precede and accompany HSC activation, reinforcing that fibrosis is the output of a multicellular injury-repair network rather than an HSC-autonomous process.

Neural regulation of HSC activity is increasingly recognized but remains incompletely defined. Recent experimental evidence indicates that the sensory neuropeptide calcitonin gene-related peptide (CGRP), acting through receptor activity-modifying protein 1 (RAMP1), can promote HSC activation and liver fibrosis through TGF-β1/Smad2 and YAP signaling (51). Separately, endogenous catecholamines released during sympathetic activation can engage β2-adrenergic receptors on HSCs; experimental β2-adrenergic/β-arrestin-2 signaling has been associated with changes in HSC proliferation, migration, survival, and collagen-related responses (52). Collectively, these findings support a potential neural influence on fibrogenesis but do not establish any single neuroadrenergic pathway as a dominant driver of human MASLD.

HSC effects on the brain are most plausibly indirect. By modifying fibrosis, sinusoidal resistance, cytokine and chemokine production, and the hepatic extracellular environment, activated HSCs can contribute to the systemic milieu that signals at vascular and neural interfaces. Direct migration of HSCs to the brain or a specific HSC-to-neuron pathway has not been demonstrated. Recent experimental evidence indicates that extracellular vesicles released by lipid-stressed hepatocytes can transfer ferritin to HSCs and promote ROS-dependent HSC activation and fibrogenesis (53). Liver-cell communication in the perisinusoidal space is increasingly well characterized (54); however, claims that HSC-derived vesicles routinely reach the intestinal epithelium or brain and drive MASLD-associated neuroinflammation exceed current evidence.

HSC activation also involves coordinated metabolic reprogramming. Increased glycolytic and mitochondrial flux can support proliferation and matrix synthesis, but activation is not an exclusive switch to aerobic glycolysis. Recent studies have identified an HSC p63–HER2–ACC1 axis that links bioenergetic and lipogenic reprogramming to fibrogenic activation (55), as well as an SNHG11–Wnt/β-catenin–GLS axis that enhances glutaminolysis and promotes HSC activation (56). These mechanisms occur within broader remodeling of lipid, glucose, and amino-acid metabolism (57). Neutrophil extracellular traps can further promote fibrogenic reprogramming in experimental MASH (58). These pathways are mechanistically informative but remain investigational treatment targets (59).

## 3 A self-reinforcing cycle: bidirectional pathways in MASLD

The following model organizes plausible interactions without assuming that every patient traverses the same cycle. Metabolic susceptibility, adiposity, diabetes, diet, sleep, medication, alcohol exposure, psychiatric comorbidity, fibrosis stage, and socioeconomic context can alter the relative contribution of each pathway. Evidence is strongest for established metabolic and inflammatory injury mechanisms and weaker for several proposed feedback links to brain function.

### 3.1 Top-down pathway: psychosocial and neuroendocrine influences

Chronic psychosocial stress activates the hypothalamic–pituitary–adrenal (HPA) axis and sympathetic nervous system, increasing glucocorticoid and catecholamine signaling. These mediators can influence appetite, sleep, physical activity, adipose lipolysis, glucose homeostasis, gut motility, mucosal perfusion, immune-cell trafficking, and epithelial repair (60, 61). Behavioral pathways are equally important: stress and mood disorders can worsen dietary quality, reduce activity, impair adherence, and alter alcohol exposure. Depression and anxiety may also develop as consequences of chronic illness, obesity, socioeconomic adversity, and reduced quality of life (8). Direct longitudinal evidence that psychological stress initiates MASLD through intestinal barrier failure remains limited.

Once microbial products reach portal blood, inflammatory signaling can amplify insulin resistance. Gut-derived LPS and other microbial signals activate TLR4-dependent inflammatory pathways in hepatic and metabolic tissues, including NF-κB signaling (37, 38). Stress-kinase-mediated inhibitory serine phosphorylation of insulin-receptor substrates and cytokine-induced suppressor-of-cytokine-signaling proteins attenuate phosphoinositide 3-kinase–protein kinase B signaling. Insulin then suppresses hepatic glucose production less efficiently. Compensatory hyperinsulinemia may nevertheless continue to activate sterol regulatory element-binding protein 1c and *de novo* lipogenesis, producing selective hepatic insulin resistance. In adipose tissue, impaired antilipolytic signaling increases fatty-acid delivery; in skeletal muscle, reduced glucose disposal increases insulin demand. Immune-cell recruitment, inflammasome activation, lipotoxic intermediates, and oxidative stress further reinforce this inflammatory–metabolic cascade (4, 46).

The top-down pathway should therefore be regarded as a potential modifier, particularly in patients with sustained stress, clinically significant mood disorders, sleep disruption, adverse health behaviors, or autonomic dysregulation. Studies should measure these exposures prospectively and distinguish endocrine, behavioral, social, and metabolic mediation rather than infer a stress pathway from a cross-sectional psychiatric diagnosis alone.

### 3.2 Bottom-up pathway: hepatic and metabolic influences on the brain

The steatotic and inflamed liver releases cytokines, chemokines, hepatokines, extracellular-matrix fragments, and altered lipid and bile acid species. These signals may affect the brain through cerebrovascular endothelial activation, BBB signaling, circumventricular organs, peripheral sensory nerves, and systemic metabolic dysfunction. Microglial activation, altered synaptic plasticity, and impaired neurogenesis are plausible downstream responses (62, 63), but much of the supporting evidence derives from obesity, acute liver failure, cirrhosis, primary neurological disease, or experimental models rather than uncomplicated MASLD (64, 65). Meningeal lymphatic vessels participate in the drainage of cerebrospinal fluid, macromolecules, and immune cells and can modulate neuroinflammatory responses (66); however, a specific causal role for meningeal lymphatic dysfunction in MASLD-associated neurological manifestations has not been demonstrated.

Bile acids illustrate the need for caution. Hydrophobic species can be cytotoxic at high concentrations, while receptor-mediated effects vary among neural, glial, vascular, intestinal, and hepatic cells (23, 67). Obesity, diabetes, sleep apnea, vascular risk, and systemic inflammation can independently influence cognition and mood (9). A 2025 neuroimaging study in MASLD reported associations among liver fat, hippocampal–orbitofrontal connectivity, and cognitive-emotional measures (68). The cross-sectional design and shared metabolic determinants support coordinated organ phenotypes but do not establish liver-to-brain causality.

Subjective cognitive complaints in noncirrhotic MASLD must also be distinguished from minimal or overt hepatic encephalopathy. In advanced cirrhosis, portosystemic shunting, impaired ammonia handling, sarcopenia, systemic inflammation, and altered cerebral osmotic regulation create a different biological setting. A multi-cohort study in cirrhosis with hepatic encephalopathy identified a microbiota-associated phenethylamine mechanism and provided important systems-level insight (69). It did not fully establish the same pathway in noncirrhotic MASLD. Similarly, neurofilament light chain has shown promise in cirrhosis with minimal hepatic encephalopathy, not as a routine MASLD biomarker (70). Medication review, sleep assessment, mood evaluation, vascular risk assessment, and objective cognitive testing remain necessary before attributing ‘brain fog’ to the axis.

### 3.3 Gut microbiota as a bidirectional amplifier

The gut microbiota receives inputs from diet, medications, intestinal transit, bile acids, stress physiology, inflammation, and host genetics (61, 71). It may amplify pathology by modifying SCFA production, bile acid transformation, tryptophan metabolism, endogenous ethanol, trimethylamine-related compounds, and inflammatory signaling (72, 73). Stress-associated microbiome changes and psychobiotic effects are biologically plausible (74), but evidence from depression or neurological disease should not be treated as direct MASLD evidence. Antibiotics and other common medications can substantially modify microbial composition and must be recorded (43).

Bottom-up effects can also reshape the intestinal ecosystem. Altered bile acid secretion, delayed transit, systemic inflammation, dietary change, and medications create ecological pressure (67). Metagenomic signatures have distinguished advanced fibrosis in cohorts studied under historical NAFLD criteria (75), and recent MASLD studies have integrated microbial features with host genetic variants (76). However, signatures vary across geography and analytical platforms. Relative-abundance sequencing can generate compositional artifacts; shotgun metagenomics predicts capacity but not necessarily transcription or metabolite flux. Genetic associations and polygenic risk may further modify hepatic injury independently of the microbiome (77, 78).

Functional redundancy is therefore central: different taxa can produce similar metabolites, whereas the same organism can behave differently according to substrate availability, neighboring species, intestinal oxygen tension, and host immunity. Taxonomic ‘dysbiosis’ should not be treated as a universal disease state. Quantitative methods, harmonized collection, external validation, and linkage to clinically relevant liver outcomes are required before microbial profiles can define a treatment-responsive MASLD endotype (79).

### 3.4 Integrated model and evidence boundaries

The proposed cycle begins with interacting metabolic, behavioral, and psychosocial pressures rather than a single trigger. Neuroendocrine activation and adverse behaviors may impair metabolic and intestinal homeostasis. Barrier dysfunction and dysbiosis may increase portal microbial signaling, aggravating hepatic inflammation and insulin resistance. The liver and intestine then modify the circulating inflammatory and metabolic environment, potentially affecting vascular and neural function. Fatigue, mood symptoms, and cognitive complaints may reduce activity, worsen sleep and diet, and increase perceived stress, thereby reinforcing upstream drivers. Experimental combined MASH-diet and alcohol models illustrate how distinct insults can converge on NLRP3–IL-1β signaling (80), but such models do not reproduce every human phenotype.

**Figure 2:**
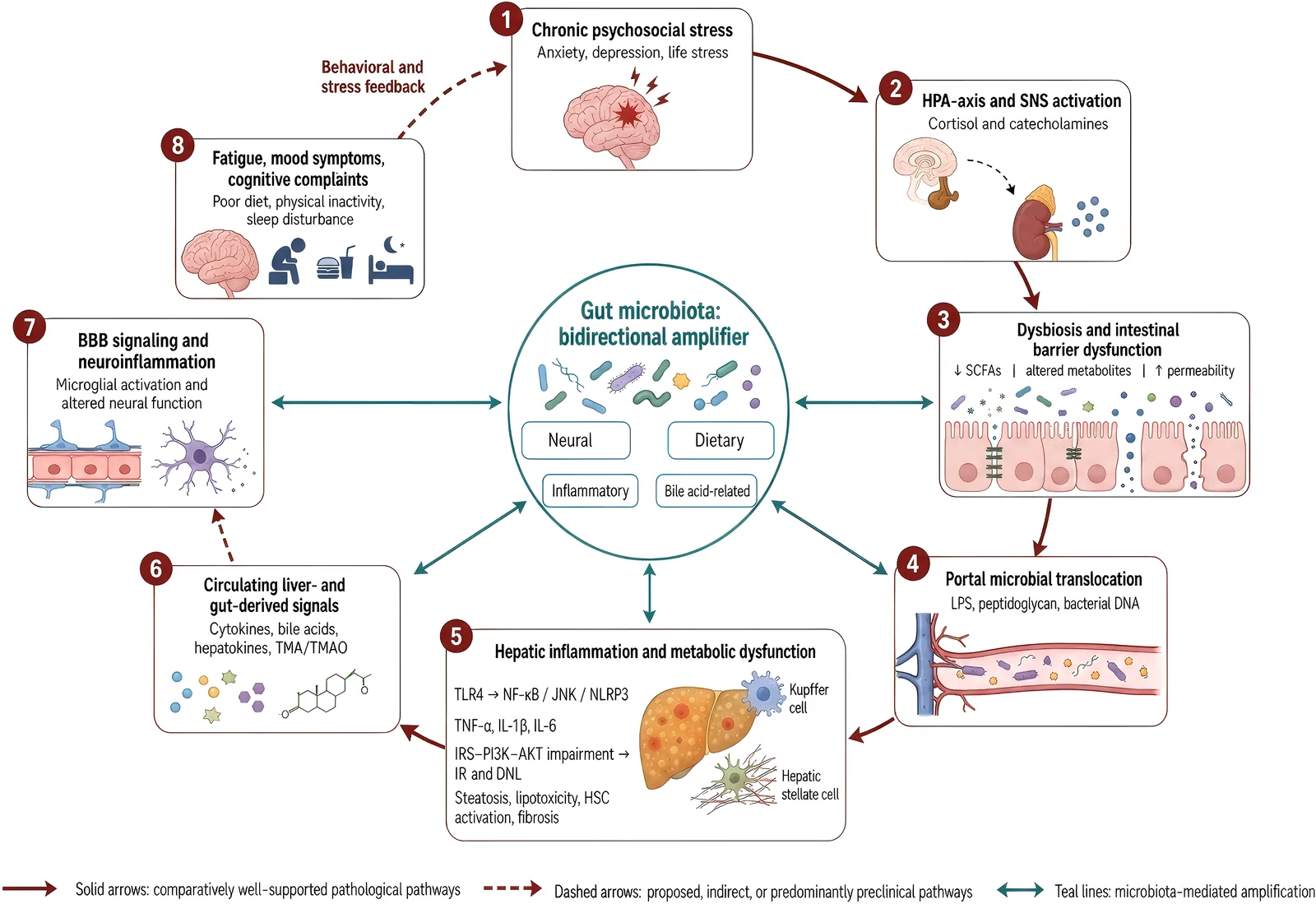
Self-reinforcing liver–gut–brain cycle in metabolic dysfunction-associated steatotic liver disease. Chronic psychosocial stress activates the HPA axis and SNS, increasing glucocorticoid and catecholaminergic signaling. These changes may affect intestinal motility, mucosal immunity, microbial composition, and epithelial integrity. Dysbiosis and barrier dysfunction reduce beneficial microbial outputs, including SCFAs, while increasing permeability and portal exposure to LPS, peptidoglycan, and bacterial DNA. In the liver, microbial signals activate pattern-recognition receptors and NF-κB, JNK, and NLRP3-associated pathways. Cytokine production can impair IRS–PI3K–AKT signaling and contribute to IR, DNL, steatosis, lipotoxicity, Kupffer-cell activation, HSC activation, and fibrosis. Liver- and gut-derived cytokines, bile acids, hepatokines, and metabolites may influence BBB function, microglial activation, and neuroinflammation. Fatigue, mood symptoms, and cognitive complaints may subsequently worsen activity, diet, sleep, and perceived stress. The microbiota acts as a bidirectional amplifier of neural, dietary, inflammatory, and bile acid-related inputs. Solid arrows indicate comparatively well-supported pathways; dashed arrows indicate proposed, indirect, or predominantly preclinical mechanisms; teal lines indicate microbiota-mediated amplification. Sections 3.3 and 3.4 explain the microbiota-dependent amplification and the evidentiary limits of the integrated cycle. AKT, protein kinase B; BBB, blood–brain barrier; DNL, *de novo* lipogenesis; DNA, deoxyribonucleic acid; HPA, hypothalamic–pituitary–adrenal; HSC, hepatic stellate cell; IL-1β, interleukin-1 beta; IL-6, interleukin-6; IR, insulin resistance; IRS, insulin receptor substrate; JNK, c-Jun N-terminal kinase; LPS, lipopolysaccharide; MASLD, metabolic dysfunction-associated steatotic liver disease; NF-κB, nuclear factor kappa B; NLRP3, NOD-, LRR- and pyrin domain-containing protein 3; PI3K, phosphoinositide 3-kinase; SCFAs, short-chain fatty acids; SNS, sympathetic nervous system; TLR4, Toll-like receptor 4; TMA, trimethylamine; TMAO, trimethylamine N-oxide; TNF-α, tumor necrosis factor alpha.

A vicious-cycle model is useful only if it remains falsifiable. Coexisting organ phenotypes are insufficient; temporal ordering, dose–response relationships, pathway specificity, mediation, and reversibility should be tested. **Figure 2** distinguishes comparatively well-supported metabolic and inflammatory pathways from proposed or predominantly preclinical feedback links. It should not be interpreted as evidence that all patients follow the complete cycle.

## 4 Translational implications

Translation requires separating tools that improve established hepatic risk stratification from research methods that interrogate inter-organ communication. No validated test currently measures the liver–gut–brain axis as a single clinical entity, and no treatment should be selected solely on the basis of an unvalidated axis subtype.

### 4.1 Methodological advances

#### 4.1.1 Neuroimaging and organ-level phenotyping

Magnetic resonance imaging-derived proton density fat fraction and elastography quantify hepatic steatosis and stiffness, while structural, diffusion, perfusion, and functional brain MRI can characterize gray matter and network connectivity (81). The 2025 MASLD neuroimaging study provides a direct example of combined hepatic and brain phenotyping (68). Preclinical molecular positron-emission tomography targeting C-C chemokine receptor type 2 (CCR2) and cluster of differentiation 163 (CD163) can track changes in recruited and resident hepatic macrophage populations during MASH progression and regression (82). However, these approaches remain experimental, and neither molecular nor total-body imaging has validated a specific MASLD liver–brain signature. Robust protocols require synchronized acquisition, validated fibrosis assessment, prespecified brain outcomes, motion correction, objective neuropsychological testing, and adjustment for obesity, diabetes, sleep apnea, vascular disease, and medication use.

#### 4.1.2 Multi-omics and mechanistic studies

Metagenomics, metabolomics, transcriptomics, and experimental perturbation have generated testable hypotheses. **Table 1** presents representative studies in a structured format, as evidence contexts are not equivalent. The human MASLD imaging study directly assessed brain outcomes; the MAFLD metabolomics study used a related but distinct diagnostic framework; the hepatic encephalopathy study involved advanced cirrhosis; and the intestinal-gene and probiotic studies were predominantly experimental. These distinctions prevent over-extrapolation.

**Table 1:** Representative studies informing the liver–gut–brain axis.

Evidence context	Approach	Principal finding	Relevance	Major limitation	Ref.
Human MASLD	Structural and resting-state functional MRI	Liver fat was associated with hippocampal–orbitofrontal alterations and cognitive-emotional measures.	Direct human liver–brain phenotype	Cross-sectional; modest sample; shared metabolic confounding	(68)
Human study using MAFLD framework	Longitudinal metabolomics and cognitive trajectories	Serum metabolites partially mediated an association between the MAFLD framework and cognitive decline.	Potential metabolic mediation	MAFLD is not identical to MASLD; residual confounding and mediation assumptions	(83)
Cirrhosis with hepatic encephalopathy	Multi-cohort metagenomics and mechanistic validation	Identified a possible microbial phenethylamine pathway associated with cognitive dysfunction.	Microbiota–metabolite–brain mechanism	Advanced cirrhosis/HE; not directly generalizable to noncirrhotic MASLD	(69)
Experimental MASH/gut–liver model	Genetic and metabolic perturbation of intestinal TM6SF2	Supported a causal intestinal lipid-handling pathway influencing steatohepatitis.	Mechanistic intestinal control of hepatic injury	Predominantly experimental; human effect size uncertain	(40)
Mouse liver-injury model	Akkermansia muciniphila intervention	Improved liver injury and cognitive readouts with BDNF/serotonin-pathway changes.	Potential gut–liver–brain modulation	Animal model; strain, dose, and disease context limit translation	(84)
Chronic-stress mouse model	Bifidobacterium pseudonumeratum W112, 16S sequencing, hepatic metabolomics	Altered microbial composition, liver injury, and depressive-like behavior.	Stress–microbiota–liver interactions	Not a MASLD model; behavioral and taxonomic endpoints are model dependent	(85)

16S rRNA, 16S ribosomal RNA gene; BDNF, brain-derived neurotrophic factor; HE, hepatic encephalopathy; MAFLD, metabolic dysfunction-associated fatty liver disease; MASH, metabolic dysfunction-associated steatohepatitis; MASLD, metabolic dysfunction-associated steatotic liver disease; MRI, magnetic resonance imaging; TM6SF2, transmembrane 6 superfamily member 2. The table separates direct human MASLD evidence from studies in MAFLD, cirrhosis, or animal models.

Multi-omics integration is most informative when biospecimens and organ phenotypes are collected at aligned time points. Stool metagenomics should be paired with dietary records, medication exposure, stool consistency, microbial load, and metabolite measurements. Plasma metabolomics requires standardized fasting state, processing time, storage, and renal-function assessment. Liver transcriptomic or spatial data can identify cellular sources but are usually obtained from selected biopsy populations. Brain imaging adds another high-dimensional layer with its own motion, multiplicity, and reproducibility challenges. Statistical integration should therefore include prespecified hypotheses, independent validation, correction for multiple testing, transparent handling of missing data, and sensitivity analyses for adiposity and diabetes. Mechanistic follow-up is required because cross-platform correlation can identify coordinated variation without establishing directionality.

#### 4.1.3 Non-invasive, digital, and analytical assessment

Non-invasive liver tests identify patients at risk of advanced fibrosis, but no score measures axis function. Digital measures of sleep, activity, heart-rate variability, diet, mood, and treatment adherence can add longitudinal context. A prescription digital therapeutic has shown feasibility for reducing liver fat (86), but short-duration, uncontrolled, or feasibility designs cannot establish equivalence to proven hepatic interventions. Artificial-intelligence models may integrate multimodal data, but external validation, calibration, transparency, privacy, and algorithmic-bias assessment are prerequisites. Parallel improvement in liver fat and brain or symptom outcomes after weight loss would still not prove direct inter-organ causality.

### 4.2 Candidate biomarkers and endotyping

Candidate markers can be organized by biological domain, but few are specific to MASLD and none defines a validated liver–gut–brain endotype. Intestinal fatty acid-binding protein has been associated with permeability and steatosis in a prospective MASLD cohort (41), while microbial translocation markers have been associated with fibrosis in selected populations (42). Fibroblast growth factor 21 reflects hepatokine and metabolic stress but overlaps with obesity and diabetes (87). Neurofilament light chain data arise mainly from cirrhosis with hepatic encephalopathy (70). These context differences are summarized in **Table 2**.

**Table 2:** Candidate biomarkers of liver–gut–brain axis dysfunction in MASLD.

Domain	Representative markers	Biological meaning	Potential relevance	Current limitations	Ref.
Intestinal injury/permeability	I-FABP; sugar permeability tests; tight-junction fragments	Enterocyte injury or altered barrier integrity	Emerging human MASLD data	Limited specificity; methods assess different processes	(41)
Microbial translocation	LPS, LBP, sCD14, bacterial DNA	Exposure to microbial products and innate immune activation	Associated with fibrosis in selected cohorts	Pre-analytical contamination; transient exposure; comorbidity confounding	(37, 42)
Microbial metabolites	SCFAs, indoles, TMAO, PAGln, imidazole propionate	Microbial metabolic output with immune, vascular, and neural effects	Potential functional readout beyond taxonomy	Diet-, kidney-, drug-, and platform-dependent; no validated panel	(26, 29–30)
Bile acid signaling	Primary/secondary ratios, FGF19, 7α-hydroxy-4-cholesten-3-one	Enterohepatic cycling and FXR-related signaling	Potential gut–liver functional signature	Circadian and postprandial variation; context-dependent receptor effects	(90–91)
Liver injury/metabolic stress	ALT, AST, CK-18 fragments, FGF21	Hepatocyte injury and metabolic adaptation	Useful within multimodal hepatic assessment	Not specific to axis dysfunction; normal ALT does not exclude fibrosis	(6, 87)
Fibrosis/ECM remodeling	FIB-4, ELF components, PRO-C3	Fibrosis risk and extracellular-matrix turnover	Closer to validated MASLD risk stratification	Measures hepatic fibrosis rather than gut or brain involvement	(6, 92)
Systemic inflammation	hs-CRP, IL-6, TNF-α, IL-1β, IL-1RA	Systemic inflammatory tone	Potential link between metabolic inflammation and symptoms	Highly nonspecific and affected by obesity, infection, and comorbidity	(44, 47)
Neuroaxonal/glial injury	NfL, GFAP, S100B	Neuroaxonal injury or glial activation	Research markers for neurological involvement	Evidence mainly from cirrhosis/HE or neurological disease	(70)
Neuroendocrine/autonomic	Cortisol profiles, catecholamines, heart-rate variability	HPA-axis and autonomic activity	May characterize stress-related physiology	Circadian effects, medications, fitness, sleep, and device variability	(61)

ALT, alanine aminotransferase; AST, aspartate aminotransferase; CK-18, cytokeratin-18; DNA, deoxyribonucleic acid; ECM, extracellular matrix; ELF, enhanced liver fibrosis; FGF19/21, fibroblast growth factor 19/21; FIB-4, Fibrosis-4 index; FXR, farnesoid X receptor; GFAP, glial fibrillary acidic protein; HE, hepatic encephalopathy; HPA, hypothalamic–pituitary–adrenal; hs-CRP, high-sensitivity C-reactive protein; I-FABP, intestinal fatty acid-binding protein; IL, interleukin; IL-1RA, interleukin-1 receptor antagonist; LBP, LPS-binding protein; LPS, lipopolysaccharide; MASLD, metabolic dysfunction-associated steatotic liver disease; NfL, neurofilament light chain; PAGln, phenylacetylglutamine; PRO-C3, N-terminal propeptide of type III collagen; S100B, S100 calcium-binding protein B; sCD14, soluble cluster of differentiation 14; SCFAs, short-chain fatty acids; TMAO, trimethylamine N-oxide; TNF-α, tumor necrosis factor alpha. All listed biomarkers remain investigational for axis endotyping and are not validated for routine MASLD treatment selection.

Metabolomic panels may capture functional interactions but are sensitive to diet, fasting state, renal function, medication, circadian variation, and analytical platform. A longitudinal study explicitly used the MAFLD diagnostic framework and identified candidate metabolites mediating cognitive trajectories (83). Recent studies have also linked mental health, MASLD, and circulating metabolites (88) and described altered tryptophan–kynurenine profiles (89). These findings are hypothesis generating. Bile acid profiles in advanced fibrosis (90, 91), host genetic variants (76, 77), and polygenic scores (78) may improve biological characterization but do not currently select axis-targeted therapy.

Biomarker development should specify the intended use—fibrosis detection, mechanistic subgrouping, symptom prediction, or response monitoring—and demonstrate reproducibility, calibration, discrimination, and incremental value beyond established clinical variables. Fibrosis markers such as the N-terminal propeptide of type III collagen (PRO-C3) have clearer hepatic prognostic relevance than most axis candidates but measure matrix remodeling rather than inter-organ communication (92). A statistical association does not make a marker a mediator, treatment target, or surrogate endpoint.

A clinically useful panel would need to outperform simpler alternatives and change management. Discovery studies should report analytical variation, batch correction, missingness, and the influence of age, sex, ethnicity, body composition, renal function, diet, and medication. External validation must reproduce both direction and effect size in a population with a similar intended use. Longitudinal studies should establish within-person stability and responsiveness, while intervention studies should determine whether change tracks a meaningful hepatic or patient-centered outcome. For endotyping, clusters must be reproducible across algorithms and remain informative after established fibrosis and cardiometabolic variables are included. Without these steps, increasingly complex panels risk describing population structure rather than identifying a causal or treatment-responsive axis phenotype.

### 4.3 Therapeutic strategies

#### 4.3.1 Foundational lifestyle and brain-directed care

Lifestyle intervention and management of obesity, type 2 diabetes, dyslipidemia, hypertension, sleep disorders, and cardiovascular risk remain foundational (6, 7, 93). Mediterranean-style dietary patterns have cardiometabolic and hepatic benefits, although microbiome change is only one possible mediator (94, 95). A low-glycemic Mediterranean intervention combined with physical activity altered microbiota composition in a cohort studied under historical NAFLD terminology (96). Dietary quality, aerobic and resistance exercise, reduced sedentary time, and sustained weight loss can benefit patients even when an axis mechanism is not demonstrated.

Psychological assessment is appropriate when fatigue, depression, anxiety, sleep disturbance, or cognitive complaints impair functioning. Behavioral therapy may improve coping, adherence, sleep, and quality of life; a prescription digital therapeutic has preliminary MASLD data (86), but neither should be presented as a proven antifibrotic treatment. Transcutaneous vagus nerve stimulation remains experimental. A rat study reported improvement in disease described using historical NAFLD terminology (97), whereas a sham-controlled human study in diabetes found no significant anti-inflammatory effect, including no reduction in TNF-α (98). General vagus-nerve literature supports biological plausibility (24) but not clinical efficacy in MASLD.

#### 4.3.2 Microbiota- and barrier-directed interventions

Probiotics, prebiotics, and synbiotics have produced heterogeneous changes in aminotransferases, body weight, lipids, and liver fat. A randomized controlled study reported a reduction in MRI-derived liver fat after a multispecies probiotic mixture (99), and a recent meta-analysis reported modest biochemical benefits (100). Interpretation is constrained by strain specificity, dose, duration, baseline microbiome, concomitant diet, small samples, varying diagnostic criteria, endpoint heterogeneity, and limited histological outcomes. Products should not be treated as interchangeable therapies.

Fecal microbiota transplantation (FMT) is experimental in MASLD. In a proof-of-concept randomized trial of 21 participants diagnosed under historical NAFLD criteria, allogenic FMT improved small-intestinal permeability in participants with elevated baseline permeability but did not improve hepatic proton density fat fraction or insulin resistance (101). Donor selection, pathogen transmission, long-term ecological effects, manufacturing consistency, recipient selection, and regulatory oversight remain barriers. Improved permeability alone is not evidence of histological benefit.

#### 4.3.3 Liver- and metabolism-directed pharmacotherapy

Resmetirom, a liver-directed thyroid hormone receptor beta agonist, was the first drug granted accelerated U.S. approval for adults with noncirrhotic MASH and moderate-to-advanced fibrosis, in conjunction with diet and exercise (102). MAESTRO-NASH demonstrated increased MASH resolution without worsening fibrosis and fibrosis improvement without worsening MASH (103). Monitoring should address hepatotoxicity, gallbladder-related events, thyroid status, and interactions with selected statins; confirmation of long-term clinical benefit remains ongoing.

Glucagon-like peptide-1 receptor agonists (GLP-1 RAs) have strong metabolic and weight-management evidence. An updated meta-analysis of phase 2 and phase 3 randomized trials found that GLP-1 RAs increased the likelihood of MASH resolution, reduced magnetic resonance-measured liver fat, and improved fibrosis outcomes primarily in patients with noncirrhotic MASH, although evidence for long-term liver-related clinical events remains limited (104). Earlier phase 2 semaglutide data showed increased steatohepatitis resolution without significant fibrosis improvement under historical NASH terminology (105). In the phase 3 ESSENCE interim analysis, semaglutide 2.4 mg increased MASH resolution and fibrosis improvement at 72 weeks (106). In August 2025, the U.S. Food and Drug Administration granted accelerated approval to injectable Wegovy for adults with noncirrhotic MASH and moderate-to-advanced fibrosis (107). Cardiovascular benefit in people with overweight or obesity is supported by SELECT (108), but gastrointestinal adverse effects, gallbladder disease, cost, and persistence remain important.

Dual and triple incretin agents should be distinguished from approved MASH therapies. Tirzepatide increased MASH resolution without worsening fibrosis in SYNERGY-NASH but is not specifically approved for MASH (109). Sodium–glucose cotransporter 2 inhibitors provide glycemic, cardiovascular, and renal benefits in appropriate patients with type 2 diabetes and may also reduce liver fat. In a 2023 randomized study of patients with early-stage type 2 diabetes and NAFLD, empagliflozin reduced intrahepatic lipid content more than sitagliptin, although between-group changes in tissue-specific insulin sensitivity were not significant (110). Definitive histological and antifibrotic evidence remains insufficient.

FXR agonists remain investigational for MASH. The REGENERATE trial of obeticholic acid met a fibrosis-improvement endpoint but was limited by pruritus and increased low-density lipoprotein cholesterol (111). The U.S. Food and Drug Administration issued a complete response letter in 2023, and obeticholic acid is not approved for MASH/NASH. REGENERATE is an obeticholic-acid trial rather than a GLP-1 receptor agonist trial. Cilofexor and other nonsteroidal FXR agonists have phase 2 data (112), but safety, tolerability, and clinical-outcome evidence remain insufficient. More broadly, anti-inflammatory and antifibrotic combination strategies remain under evaluation (59, 113).

Drug selection should occur within a risk-stratified pathway rather than according to a presumed axis subtype. Histological or validated non-invasive evidence of clinically significant fibrosis, cirrhosis status, obesity, type 2 diabetes, cardiovascular and renal disease, thyroid disease, concomitant medication, reproductive considerations, adverse-effect profile, local regulatory criteria, cost, and patient preference all influence treatment. Resmetirom and semaglutide have different mechanisms, eligibility criteria, and monitoring considerations; neither removes the need for lifestyle and cardiometabolic management. When incretin-based therapy or an SGLT2 inhibitor is prescribed primarily for an approved metabolic indication, improvement in aminotransferases or liver fat should not be equated automatically with fibrosis regression. Head-to-head and combination trials, durability data after discontinuation, and clinical-outcome studies are still required.

#### 4.3.4 Management of psychiatric comorbidity

Antidepressant choice should be individualized according to depressive phenotype, obesity, appetite and weight trajectory, sleep disturbance, diabetes risk, hepatic impairment, drug interactions, previous response, bipolar or psychotic features, suicidality, and patient preference. Tricyclic antidepressants or mirtazapine should not be categorically excluded, and selective serotonin or serotonin–norepinephrine reuptake inhibitors should not be presented as universally preferred without MASLD-specific comparative evidence. Weight effects vary within and across drug classes (114). Treating depression and anxiety may improve adherence, lifestyle behavior, and quality of life, but direct evidence that antidepressants modify MASLD histological progression is limited (8).

**Table 3** summarizes the mechanism, evidence or regulatory status, direct supporting references, and principal limitations of each therapeutic category.

**Table 3:** Therapeutic strategies relevant to the liver–gut–brain axis in MASLD.

Target	Intervention	Axis-related rationale	Evidence/regulatory status	Key limitations	Ref.
Lifestyle/weight	Mediterranean-style diet, activity, weight reduction	Improves adiposity, insulin sensitivity, and cardiometabolic risk; may modify microbiota	Foundational guideline-supported care	Adherence, access, variable response; microbiome mediation not established	(6, 93, 95)
Behavioral	Psychological therapy and structured self-management	May reduce distress and improve sleep, adherence, and behavior	Supportive care; not an established antifibrotic treatment	Limited MASLD-specific hepatic outcomes	(8, 86)
Neuromodulation	tVNS	Proposed cholinergic anti-inflammatory and autonomic effects	Experimental; no MASLD efficacy trial; diabetes inflammation study negative	Parameters, sham effects, durability, uncertain hepatic endpoints	(97–98)
Microbiota	Probiotics/prebiotics/synbiotics	Potential barrier and microbial-metabolite modulation	Heterogeneous small RCTs/meta-analyses; no definitive histological benefit	Strain, dose, duration, baseline microbiome, endpoint heterogeneity	(99–100)
Microbiota	FMT	Ecosystem-level microbial transfer	Experimental; one small proof-of-concept MASLD/NAFLD trial	No liver-fat or IR benefit; donor, safety, manufacturing, regulatory issues	(101)
Liver	Resmetirom	THR-β activation reduces hepatic lipids and improves atherogenic lipids	Accelerated approval for selected adults with noncirrhotic MASH and F2–F3 fibrosis	Long-term outcomes pending; hepatotoxicity, gallbladder events, interactions	(102–103)
Metabolic/liver	Semaglutide 2.4 mg	Weight loss, improved insulin resistance, and direct/indirect hepatic effects	Accelerated approval for selected adults with noncirrhotic MASH and moderate-to-advanced fibrosis	GI adverse effects, gallbladder disease, cost, persistence	(106–107)
Metabolic	Dual/triple incretin agents, including tirzepatide	Weight loss and multi-receptor metabolic signaling	Promising phase 2 MASH evidence; not specifically approved for MASH	Long-term histological and clinical outcomes; access	(109)
Metabolic	SGLT2 inhibitors	Glucosuria, weight and glycemic effects; possible liver-fat reduction	Use according to diabetes/heart/kidney indications; supportive imaging evidence	Insufficient definitive histological evidence; class adverse effects	(110)
Bile acid	FXR agonists, including obeticholic acid	Modulate bile acid synthesis and metabolic/fibrotic signaling	Investigational for MASH; obeticholic acid is not approved	Pruritus, LDL-C increase, safety/tolerability and regulatory limitations	(111–112)

F2–F3, fibrosis stages 2–3; FMT, fecal microbiota transplantation; FXR, farnesoid X receptor; GI, gastrointestinal; IR, insulin resistance; LDL-C, low-density lipoprotein cholesterol; MASH, metabolic dysfunction-associated steatohepatitis; MASLD, metabolic dysfunction-associated steatotic liver disease; NAFLD, non-alcoholic fatty liver disease; RCT, randomized controlled trial; SGLT2, sodium–glucose cotransporter 2; THR-β, thyroid hormone receptor beta; tVNS, transcutaneous vagus nerve stimulation. Regulatory status is current to July 15, 2026 and may differ across jurisdictions.

### 4.4 Clinical integration, limitations, and research priorities

#### 4.4.1 Clinical integration and practical implications

The 2024 EASL–EASD–EASO guideline recommends case-finding for advanced fibrosis in people with cardiometabolic risk, particularly type 2 diabetes or obesity with additional risk factors. A staged pathway commonly begins with the Fibrosis-4 index and proceeds, when indicated, to elastography or another validated non-invasive test (6). The AASLD guidance similarly emphasizes risk stratification and management of metabolic comorbidity (7). Treatment selection should be based on fibrosis risk, cirrhosis status, metabolic disease, contraindications, patient preference, regulatory eligibility, monitoring requirements, and access.

Clinically, fibrosis risk and symptom burden should be assessed in parallel because fatigue, mood, sleep, and cognitive complaints are important but do not reliably indicate histological severity. The liver–gut–brain axis is currently most useful as an organizing framework rather than as a validated diagnostic or treatment-selection endotype; accordingly, stool sequencing, brain-derived biomarkers, and autonomic measures should not guide routine treatment selection. Sustained lifestyle intervention, weight management, and optimal treatment of diabetes, cardiovascular risk, sleep disorders, and psychiatric comorbidity remain the foundation of care. For eligible adults with noncirrhotic MASH and moderate-to-advanced fibrosis, resmetirom or semaglutide may be considered where locally approved; treatment should be individualized according to fibrosis status, comorbidities, contraindications, monitoring requirements, access, and patient preference. Probiotics, fecal microbiota transplantation, and transcutaneous vagus nerve stimulation remain investigational and should not replace evidence-based hepatology and cardiometabolic care.

#### 4.4.2 Limitations of the current evidence

Three limitations define the field. First, the evidence base is uneven: many proposed pathways are supported mainly by animal studies, studies in cirrhosis, or evidence from other inflammatory and neurological diseases rather than prospective MASLD cohorts. Second, variation in diet, geography, medication use, psychiatric comorbidity, genetics, and microbiome composition limits generalizability and makes universal signatures unlikely. Third, hepatic, microbial, inflammatory, autonomic, and neurobehavioral endpoints remain heterogeneous.

#### 4.4.3 Research priorities

Future studies should align specimen collection and phenotyping, prespecify disease stage and evidence context, use credible controls, distinguish biomarker change from clinical benefit, and include both hepatic and patient-centered outcomes. External replication and causal mediation or perturbation designs are essential.

An efficient research pathway would recruit participants across steatosis, MASH, and fibrosis stages and longitudinally record alcohol exposure and cardiometabolic treatments. Diet, stool, blood, imaging, autonomic data, and symptom assessments should be collected within predefined windows. Nested studies could then test whether a candidate microbial metabolite, bile acid signal, hepatokine, or autonomic measure mediates changes in both hepatic and neurobehavioral outcomes. Trials of probiotics, FMT, or neuromodulation require credible sham or placebo controls, standardized products or stimulation parameters, safety surveillance, and liver endpoints beyond aminotransferases. Core outcome sets and data sharing would improve cross-study comparability, and replication across geographic regions is essential for microbiome findings. Inclusion of diverse metabolic, psychiatric, and sociodemographic profiles will determine whether proposed mechanisms are generalizable or confined to selected subgroups.

## 5 Conclusions and future perspectives

The liver–gut–brain axis provides a coherent framework for investigating how metabolic dysfunction, intestinal barrier integrity, microbial ecology, hepatic inflammation, and neurobehavioral symptoms may interact in MASLD. It should not displace established pathophysiology or clinical priorities. Fibrosis risk stratification, sustained lifestyle intervention, and optimal management of obesity, diabetes, cardiovascular risk, and other comorbidities remain central. Approved pharmacotherapies now offer additional options for selected adults with noncirrhotic MASH and moderate-to-advanced fibrosis. Microbiota-based endotyping, FMT, tVNS, and neurological biomarker panels remain investigational.

Progress will require longitudinal human cohorts and intervention trials integrating standardized liver phenotyping with diet, medication exposure, intestinal permeability, microbial function, systemic inflammation, autonomic measures, and validated neuropsychological outcomes. Causal mediation, tracer, and perturbation designs should distinguish shared cardiometabolic confounding from true inter-organ signaling. The immediate clinical value of the axis is organizational: it encourages coordinated assessment of hepatic, metabolic, intestinal, neurological, and psychosocial determinants. Multidisciplinary care may improve symptom management, adherence, and quality of life, but it should complement rather than replace evidence-based hepatology and cardiometabolic care.

Careful evidence grading will be essential as this field moves from conceptual integration toward clinically testable, patient-specific hypotheses.
